# Transmission of *Metarhizium anisopliae* and *Beauveria bassiana* to adults of *Kuschelorhynchus macadamiae* (Coleoptera: Curculionidae) from infected adults and conidiated cadavers

**DOI:** 10.1038/s41598-021-81647-0

**Published:** 2021-01-26

**Authors:** Kim Khuy Khun, Gavin J. Ash, Mark M. Stevens, Ruth K. Huwer, Bree A. L. Wilson

**Affiliations:** 1 Faculty of Agronomy, Royal University of Agriculture, Dangkor District, P.O. Box 2696, Phnom Penh, Cambodia; 2 Centre for Crop Health, Institute for Life Sciences and the Environment, University of Southern Queensland, Toowoomba, QLD 4350 Australia; 3 NSW Department of Primary Industries, Yanco Agricultural Institute, Yanco, NSW 2703 Australia; 4 Graham Centre for Agricultural Innovation, NSW Department of Primary Industries, Charles Sturt University, Wagga Wagga, NSW 2650 Australia; 5 NSW Department of Primary Industries, Wollongbar Primary Industries Institute, Wollongbar, NSW 2477 Australia

**Keywords:** Zoology, Entomology, Microbiology, Applied microbiology, Fungi

## Abstract

*Kuschelorhynchus macadamiae* is a major pest of macadamias in Australia, causing yield losses of up to 15%. Our previous studies have shown the weevil is susceptible to *Beauveria bassiana* and *Metarhizium anisopliae*. The aim of this study was to investigate horizontal transmission of both fungal species to healthy weevils from both infected adults and weevil cadavers. In a confined environment the mortality of healthy adults caused by the transmission of conidia from live fungus-infected adults was < 50%. Under similar experimental conditions, the mortality of healthy adults reached 100% when exposed to conidiated cadavers. However, when conidiated cadavers were used in more spacious environments (insect cages), the mortality of adults was < 80%. Using scanning electron microscopy, it was observed that all healthy adults had conidia attached to all external parts of the body. This suggests that although the conidia were readily transferred to the adults, the lower mortality in the larger insect cages could be the result of an unfavourable environmental factor such as low humidity. The presence of conidia attached to all the adults indicated that they did not show any discriminatory behaviour such as avoidance of conidiated cadavers infected by these two fungal species. The results from this study show that there is potential for enhanced control of adult *K. macadamiae* via transmission from either fungus-infected adults or conidiated cadavers and this could strengthen sustainable pest management in macadamias.

## Introduction

Macadamia seed weevil, *Kuschelorhynchus macadamiae* Jennings and Oberprieler, formerly known as *Sigastus* weevil^1^, is a native Australian insect which was initially found in macadamias (*Macadamia integrifolia* Maiden and Betche and *M. tetraphylla* L.A.S. Johnson) on the Atherton Tablelands, Queensland^2^ in 1994 and later in the Northern Rivers, New South Wales (NSW)^3,4^. This weevil is a major pest of macadamias at the nut development stage^3,4^ with the female weevil ovipositing inside the husk of the macadamias when they are about 10 mm in diameter, and inducing premature nut drop between the months of September and December each year^2,5^. This premature nut drop has been estimated to lead to approximately AU$ 15 million worth of lost production^6^. Adults also feed on young leaves and completely remove the bark from seedlings, leading to plant death within a few days (K. K. Khun, personal observation).

The entomopathogenic fungi, *Beauveria bassiana* (Bals.-Criv.) Vuill. (Hypocreales: Cordycipitaceae) and *Metarhizium anisopliae* (Metschn.) Sorokin (Hypocreales: Clavicipitaceae) have cosmopolitan distributions^7,8^ and are commonly isolated from insects and soil using selective media and insect baits (such as *Galleria mellonella* L. and *Tenebrio molitor* L.), respectively^9,10^. Various studies have shown the potential of fungal entomopathogens for controlling many economically important weevils affecting horticultural crops^11–13^. In our previous study, entomopathogenic fungi looked promising for the control of *K. macadamiae*^14^. In the laboratory *B. bassiana* strain B27 and *M. anisopliae* strain ECS1 were the most effective strains, providing better control of *K. macadamiae* than a commercial strain of *B. bassiana* (PPRI 5339) or other tested fungal strains available in Australia^14^. In addition, these strains could conidiate well on weevil cadavers^14^, indicating the possibility of horizontal infection by the entomopathogens under suitable conditions.

The natural occurrence of fungal entomopathogens on *K. macadamiae* has been documented in the Northern Rivers^6^ and at least three strains of fungal entomopathogens have been isolated from *K. macadamiae* in this region^6,14^. Their activities against *K. macadamiae* in the field were attributed to the suitability of the weather conditions, the dense canopy of the mature macadamias and the agricultural practices in the region. Some studies have suggested that conserving naturally occurring fungal entomopathogens in the field could assist with control of established pests^15,16^. As the macadamia agroecosystem is naturally suitable for fungal entomopathogens, conserving naturally occurring entomopathogens may complement inundative applications of formulated entomopathogens for the control of *K. macadamiae.*

An important aspect of using entomopathogenic fungi for controlling important insect pests in horticultural systems is the capacity of the pathogens to continue to suppress pest populations in the field after their initial application by horizontal transmission or dissemination by abiotic or biotics means^9,10^. As fungal entomopathogens may require several days to cause mortality to insects, the conidia adhering to the insect exoskeleton after application may also be transferred to other adults of the same or different species via physical contact (horizontal transmission)^17–22^. Moreover, contact with conidiated cadavers is also considered a means of on-going suppression of the pest population (horizontal infection). This is mainly due to the number of conidia on insect cadavers being at least 10 times higher than the number of conidia on fungus-infected adults^14^, and conidia on the cadavers being easily picked up by other insects^23^. The conidia present on cadavers have also been shown to be more tolerant of solar radiation under field conditions^24^. One study found that around 89% of *B. bassiana* conidia remained viable after cadavers were exposed directly to the sunlight for up to 2 weeks and around 87% of conidia remanined viable when the cadavers were shaded inside a PVC cylinder in the field for up to 20 weeks^24^. High inoculum levels and strong persistence suggest that conidia present on cadavers have the potential to suppress pest populations in the field, however, only a few studies have explored the potential for conidia transmission via physical contact with conidiated cadavers (e.g. diamondback moth, *Plutella xylostella* L.^25^, the Asian citrus psyllid, *Diaphorina citri* Kuwayama^26^, sweetpotato weevil, *Cylas formicarius* F.^17^ and the Colorado potato beetle, *Leptinotarsa decemlineata* Say^27,28^).

No previous studies have examined the transmission of entomopathogens between *K. macadamiae* individuals or the ability of conidiated cadavers to cause disease transmission in this species. Our goals in this study were to investigate and understand fungal infection in weevil populations driven by the proportion of fungus-infected adults or conidiated cadavers, and document the behaviour of adults toward conidiated cadavers killed by different fungal species.

## Results

### Horizontal transmission from fungus-infected adults to healthy adults

The mortality of all fungus-infected adults or donors (marked with red ink) including positive controls was 90–100% and 88–100% for *M. anisopliae* strain ECS1 and *B. bassiana* strain B27, respectively. The mortality of healthy adult weevils was significantly increased by higher ratios of the fungus-infected adults to healthy individuals (*P* < 0.05) and over time (*P* < 0.05) for both fungal species. A significant interaction between the ratio of the B27 infected adults and the measured times on the mortality of healthy adults was also observed (*P* < 0.05), but no significant interaction was observed between the ratio of the ECS1 infected adults and the measured times (*P* = 0.4).

**Figure 1:**
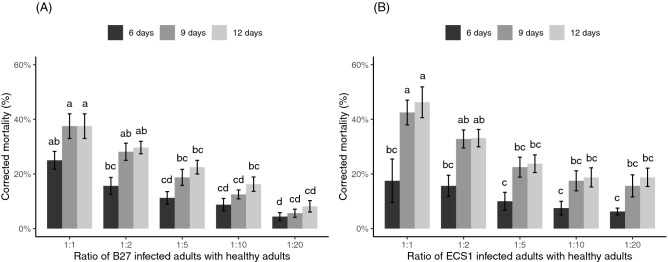
The mortality of healthy adult weevils at 6 days, 9 days and 12 days post-introduction of (**A**) B27 infected adults and (**B**) ECS1 infected adults at different ratios inside a 500 mL container. Results of multifactorial “F1-LD-F1” non-parametric analyses: (**A**) Wald-type statistics (WTS) = 126.13, df = 4, *P* < 0.001 (for ratios), WTS = 41.55, df = 2, *P* < 0.001 (for measured times), WTS = 42.19, df = 8, *P* < 0.001 (for interactions), (**B**) WTS = 34.48, df = 4, *P* < 0.001 (for ratios), WTS = 62.59, df = 2, *P* < 0.001 (for measured times), WTS = 8.31, df = 8, *P* = 0.4 (for interactions). Columns with different letters are significantly different from each other (pairwise Wilcoxon rank-sum test, *P* < 0.05). (**A**,**B**) were analysed separately. Error bars represent standard errors.

The pairwise Wilcoxon rank-sum test for multiple comparisons revealed that the highest ratio of the B27 infected adults (1:1) caused the highest mortality to healthy adults at all measured time points and was significantly higher than that observed in the three lowest ratios (1:5, 1:10, 1:20) at 6 days, 9 days and 12 days post-introduction (Fig. 1A, *P* < 0.05). For ECS1, the highest ratio of fungus-infected adults (1:1) also caused the highest mortality to healthy adults across all measured time points and was significantly higher than the mortality observed at the three lowest ratios at 9 days and 12 days post-introduction (Fig. 1B, *P* < 0.05), though there were no statistically significant differences at 6 days. Within individual ratios, only ECS1 at the 1:1 ratio produced significantly higher mortalities across time periods.

### Horizontal infection from conidiated cadavers to healthy adults in a confined environment

The mean total number of ECS1 and B27 conidia from each conidiated cadaver was 1.27 × 10^8^ and 1.35 × 10^8^ respectively. The mortality of healthy adults was significantly affected by the ratio of conidiated cadavers to healthy weevils (*P* < 0.05) and over time (*P* < 0.05) for both fungal species. No significant interaction between the ratio of the *B. bassiana* strain B27 conidiated cadavers and the measured times on the mortality of the healthy adults was found (*P* = 0.22), but a significant interaction was observed between the ratio of the *M. anisopliae* strain ECS1 conidiated cadavers and the measured times (*P* < 0.05).

**Figure 2:**
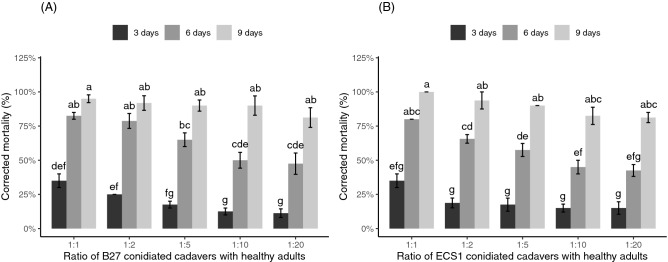
The mortality of healthy adult weevils at 3 days, 6 days and 9 days post-introduction of (**A**) B27 conidiated cadavers and (**B**) ECS1 conidiated cadavers at different ratios inside a 500 mL container. Results of multifactorial “F1-LD-F1” non-parametric analyses: (**A**) Wald-type statistics (WTS) = 47.57, df = 4, *P* < 0.001 (for ratios), WTS = 436.53, df = 2, *P* < 0.001 (for measured times), WTS = 10.64, df = 8, *P* = 0.22 (for interactions), (**B**) WTS = 93.85, df = 4, *P* < 0.001 (for ratios), WTS = 581.02, df = 2, *P* < 0.001 (for measured times), WTS = 24.45, df = 8, *P* < 0.01 (for interactions). Columns with different letters are significantly different from each other (pairwise Wilcoxon rank-sum test, *P* < 0.05). (**A**,**B**) were analysed separately. Error bars represent standard errors.

The pairwise Wilcoxon rank-sum test for multiple comparisons showed that the highest ratio of B27 conidiated cadavers to healthy adults (1:1) caused the highest mortality to healthy adults across all measured times but mortality was significantly higher than at the two lowest ratios (1:10 and 1:20) only at 3 days and 6 days post-introduction (Fig. 2A, *P* < 0.05). The highest ratio of ECS1 conidiated cadavers (1:1) caused the highest mortality to healthy adults at all measured times but in contrast to B27 mortality was significantly higher than the three lowest ratios only at 6 days post-introduction (Fig. 2B, *P* < 0.05).

### Horizontal infection from conidiated cadavers to healthy adults in an insect cage

**Figure 3:**
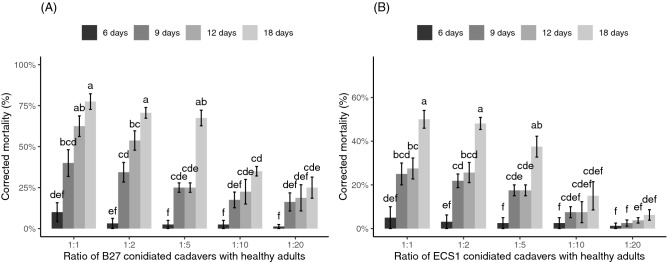
The mortality of healthy adult weevils at 6 days, 9 days 12 days and 18 days post-introduction of (**A**) B27 conidiated cadaver and (**B**) ECS1 conidiated cadaver at different ratios inside an insect cage. Results of multifactorial “F1-LD-F1” non-parametric analyses: (**A**) Wald-type statistics (WTS) = 45.42, df = 4, *P* < 0.001 (for ratios), WTS = 413.89, df = 3, *P* < 0.001 (for measured times), WTS = 72.66, df = 12, *P* < 0.001 (for interactions), (**B**) WTS = 135.16, df = 4, *P* < 0.001 (for ratios), WTS = 107.64, df = 3, *P* < 0.001 (for measured times), WTS = 97.11, df = 12, *P* < 0.001 (for interactions). Columns with different letters are significantly different from each other (pairwise Wilcoxon rank-sum test, *P* < 0.05). (**A**,**B**) were analysed separately. Error bars represent standard errors.

The mortality of healthy adults was significantly influenced by the ratio of the conidiated cadavers (*P* < 0.05), time (*P* < 0.05) and their interactions (*P* < 0.05) for both fungal species. The pairwise Wilcoxon rank-sum test for multiple comparisons showed that the highest ratio of *B. bassiana* strain B27 conidiated cadavers (ratio 1:1) caused the highest mortality to healthy adults across all measured times, and was significantly higher than the three lowest ratios (1:5, 1:10, 1:20) at 12 days and the two lowest ratios at 18 days post-introduction (Fig. 3A, *P* < 0.05). For *M. anisopliae* strain ECS1, the highest ratio of the conidiated cadavers (1:1) also caused the highest mortality to healthy adults across all measured times and was significantly higher than the ratio 1:20 at 9 days and 12 days and the two lowest ratios at 18 days post-introduction (Fig. 3B, *P* < 0.05).

### Relationships between the proportion of fungus-infected adults or conidiated cadavers to the mortality of healthy adults at 9 days post-introduction

**Figure 4:**
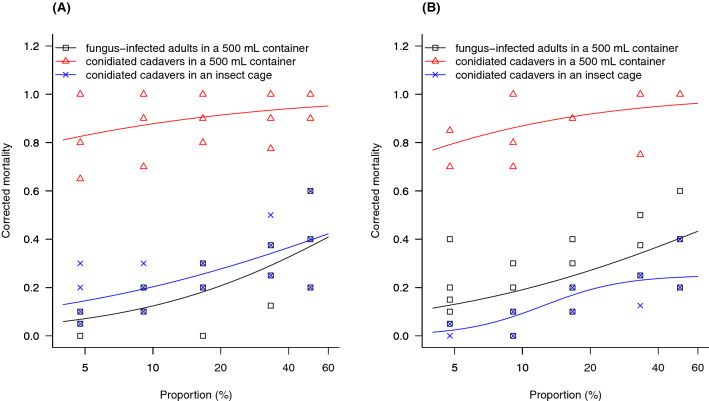
The non-linear relationship curves between the mortality of healthy adults at 9 days post-introduction and the proportion of (**A**) B27 or (**B**) ECS1 infected adults or conidiated cadavers in different experimental conditions. Individual symbol presents data of each replication and some were overlapped (bolded symbols) as shown in the figures. Models for (**A**): (∆) $y= \frac{1}{1+\mathrm{exp}(-0.56*\left(\mathrm{log}\left(x\right)-\mathrm{log}\left(0.3\right)\right))}$, (×) $y= \frac{1}{1+\mathrm{exp}(-0.58*\left(\mathrm{log}\left(x\right)-\mathrm{log}\left(102.22\right)\right))}$ and (□) $y= \frac{1}{1+\mathrm{exp}(-0.88*\left(\mathrm{log}\left(x\right)-\mathrm{log}\left(90.84\right)\right))}$, Models for (B): (∆) $y= \frac{1}{1+\mathrm{exp}(-0.75*\left(\mathrm{log}\left(x\right)-\mathrm{log}\left(0.81\right)\right))}$, (□) $y= \frac{1}{1+\mathrm{exp}(-0.65*\left(\mathrm{log}\left(x\right)-\mathrm{log}\left(90.64\right)\right))}$, and (×)$y= \frac{0.25}{1+\mathrm{exp}(-2.44*\left(\mathrm{log}\left(x\right)-\mathrm{log}\left(12.4\right)\right))}$.

For *B. bassiana* strain B27, there were positive non-linear relationships between the mortality of healthy adults and the proportion of both fungus-infected adults and conidiated cadavers (Fig. 4A). Consistent responses of the adults to either B27 infected adults or B27 conidiated cadavers in either set of experimental conditions (500 mL containers and insect cages) were found, where the best models for the three different experiments were fitted with a two-parameter log-logistic model (LL.2, Fig. 4A). The curves of these three models did not have any inflection points and this suggested that the mortality of adults continued increasing when the proportion of conidiated cadavers or fungus-infected adults increased in the population.

For *M. anisopliae* strain ECS1, there were also positive non-linear relationships between the mortality of healthy adults and the proportion of fungus-infected adults or the conidiated cadavers (Fig. 4B). The responses of the adults to either ECS1 infected adults or ECS1 conidiated cadavers in the confined environment (500 mL containers) were the same and their relationships were fitted with two-parameter log-logistic models (LL.2, Fig. 4B). However, the relationship between the mortality of adults and ECS1 conidiated cadavers in the insect cage was better described with a three-parameter log-logistic model (LL.3, Fig. 4B). The curve of the LL.3 model suggested that adult mortality reached an inflection point when the proportion of ECS1 conidiated cadavers inside the cage reached 17% (ratio 1:5). Although mortality increased with the proportion of conidiated cadavers up to 50% (ratio 1:1), based on this model the mortality of healthy adults is not expected to increase to above 27.5%.

### Scanning electron microscopy observation on the horizonal infection to healthy adults from conidiated cadavers in an insect cage

**Figure 5:**
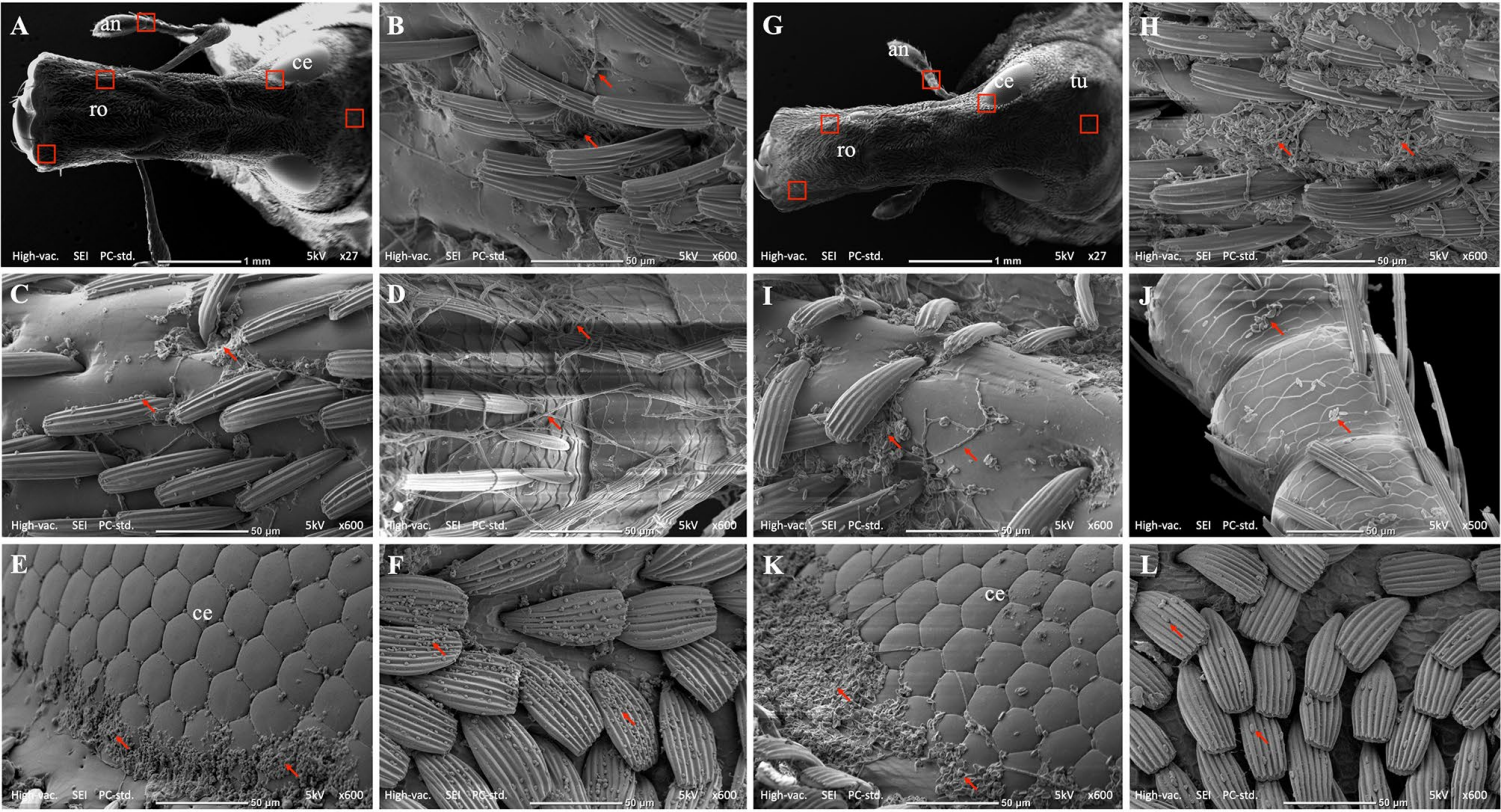
(**A**,**G**) Dorsal view of the head capsule showing antenna (an), compound eyes (ce), rostrum (ro) and tubercles (tu). Fungal conidia attached and/or germinated on (**B**,**H**) left side of rostrum, (**C**,**I**) right side of rostrum, (**D**,**J**) funicles of the antenna, (**E**,**K**) compound eyes and (**F**,**L**) scales on the head. The red arrows point at the conidia and/or the germinated conidia of B27 (**B**–**F**) and ECS1 (**H**–**L**); the red boxes in image A and G illustrates the parts of head capsule shown at higher magnification in images **B**–**F** and **H**–**L**, respectively. As there were numerous conidia either attached to, or germinated on the weevil, the arrows are used to indicate examples.

**Figure 6:**
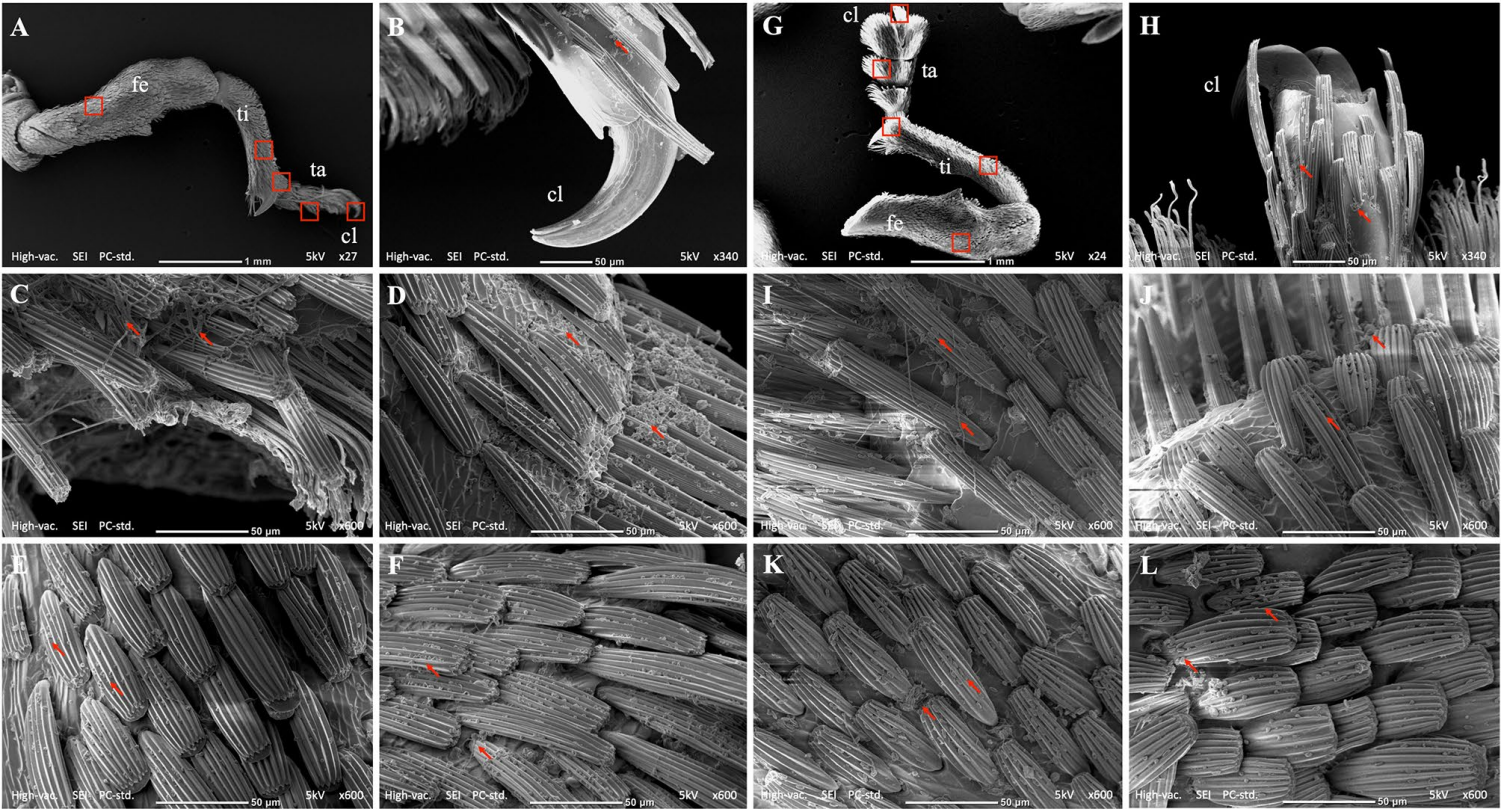
(**A**,**G**) Lateral view of the foreleg showing claws (cl), tarsus (ta), tibia (ti) and femur (fe). Fungal conidia attached and/or germinated on (**B**,**H**) claws, (**C**,**I**) tarsus, (**D**,**J**) tibial comb, (**E**,**K**) scales on the tibia and (**F**,**L**) scales on the femur. The red arrows point at the conidia and/or germinated conidia of B27 (**B**–**F**) and ECS1 (**H**–**L**); the red boxes in image A and G indicate the leg parts shown at higher magnification in images **B**–**F** and **H**–**L**, respectively. As there were numerous conidia either attached to, or germinated on the weevil, the arrows are used to indicate examples.

**Figure 7:**
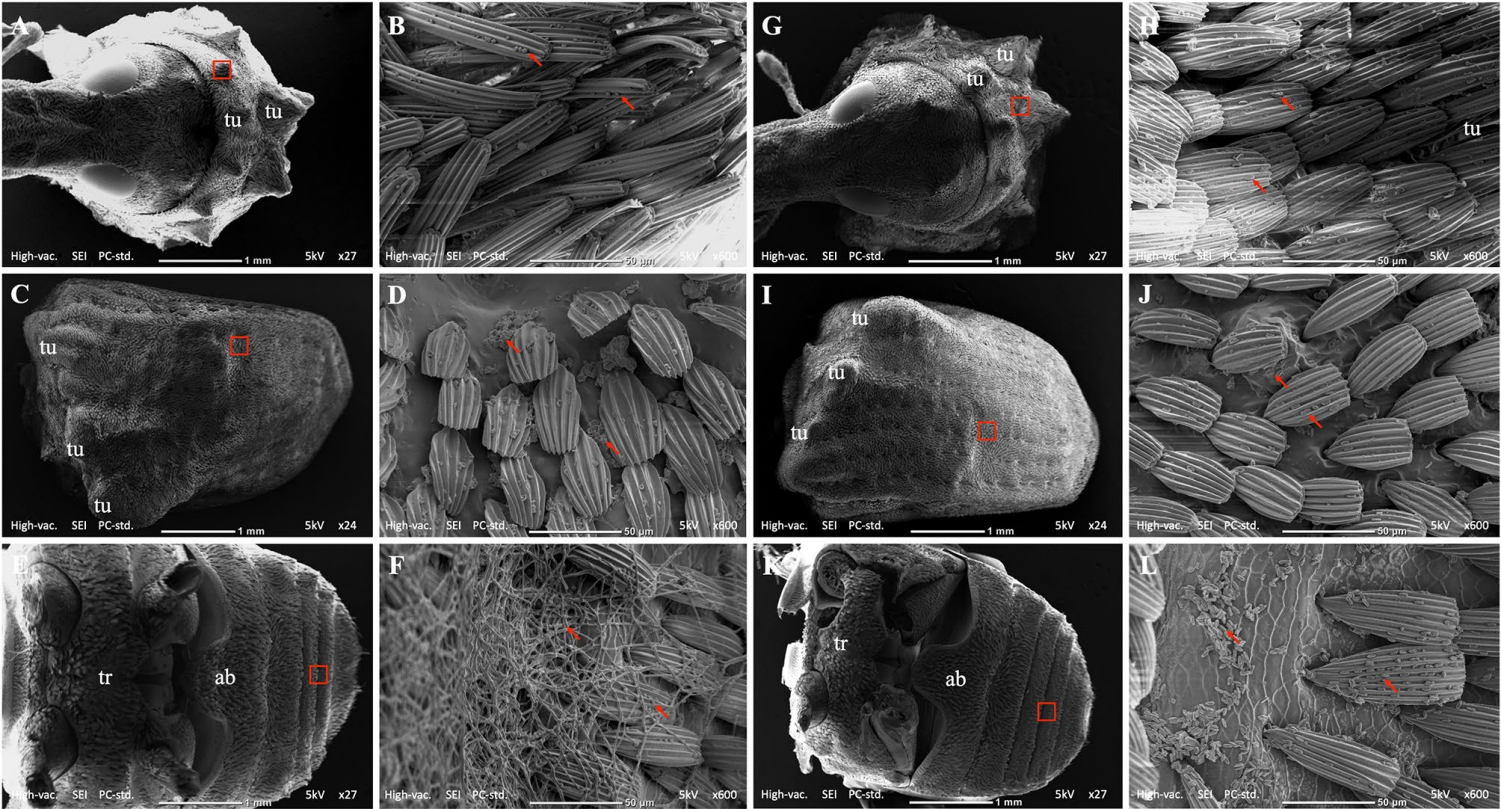
(**A**,**G**) Anterior dorsal view of the pronotum showing tubercles, (**B**,**H**) fungal conidia attached on scales around the tubercle, (**C**,**I**) Dorsal view of the elytron showing tubercles; (**D**,**J**) fungal conidia attached to the scales of the elytron, (**E**,**K**) Ventral view of the thorax (tr) and abdomen (ab), (**F**,**L**) fungal conidia germinated or attached to the scales of the abdomen. The red arrows point at the conidia or the germinated conidia of B27 (**B**,**D**,**F**) and ECS1 (**H**,**J**,**L**). As there were numerous conidia either attached to, or germinated on the weevil, the arrows are used to indicate examples. Red boxes indicate areas shown at higher magnification in other images.

All examined adults (5 adults/cage) had fungal conidia attached to all parts of their bodies at all times for both fungal species (Fig. 5, 6, 7). The number of conidia attached to the head (Fig. 5) and legs (Fig. 6) were very high (more than 400 conidia per photo at 600 × magnification) compared to other parts of the body (less than 200 conidia per photo at the same magnification) (Fig. 7). Most of the B27 conidia that were attached to hairs of the tarsal pad, tibial comb and head (particularly the rostrum and eyes) started to germinate at 6 days post-introduction, whereas the germination of ECS1 conidia was delayed until 9 days post-introduction.

## Discussion

In this study physical contact with fungus-infected adults caused low to moderate levels of mortality in initially healthy weevils. Even at the highest ratio of fungus-infected adults (1:1) in the population, the mortality of healthy adults at 12 days post-introduction was only 37.5% and 46.3%, for *B. bassiana* strain B27 and *M. anisopliae* strain ECS1, respectively. This low mortality indicates the importance of the form of the fungal conidia that are applied to the adults, with dry conidia showing improved performance relative to those sprayed as liquid formulations. Studies of conidial transmission in other insect species without the involvement of mating have shown that adults carrying conidia in a dry form could deliver infective propagules easily and cause high mortality to the recipients^22,29^. More than 60% mortality of healthy Japanese beetle (*Popillia japonica* Newman) occurred after 9 days when adults infected with conidia of *M. anisopliae* or *B. bassiana* were introduced to healthy adults at the ratio of 1:3 inside an insect cage (50 × 50 × 50 cm)^22^. Similarly, around 77% mortality of initially healthy beet webworm moth (*Spoladea recurvalis* F.) was obtained after 10 days when moths infected with conidia of *M. anisopliae* were introduced to healthy moths at the ratio 1:1 inside an insect cage (30 × 30 × 30 cm)^29^. Although dried conidia appear to be effectively transmitted and are capable of causing high mortality to the recipients^22,29^, the application of dried conidia onto crops in the field may not be viable unless they are combined with attractants of some sort. The integration of dried *B. bassiana* conidia with the sex pheromone for *C. formicarius* showed that high mortality of adults (> 90%) can be obtained in the field after three weeks of trap deployment^30^. Similarly, black vine weevil, *Otiorhynchus sulcatus* F., can be successfully controlled in the field by integrating an attractant ((Z)-2-pentenol + methyl eugenol) with *B. bassiana*^31^. Dry fungal entomopathogens have also been integrated with other attractants such as aggregation pheromones for controlling other weevil species including banana weevil, *Cosmopolites sordidus* Germar^32–34^ and red palm weevil, *Rhynchophorus ferrugineus* Olivier^35,36^.

Although some studies have shown that dried conidia can be highly effective for fungal disease transmission^22,29^, the dried conidia may also be easily removed by grooming behaviour or in the environment, and the efficacy of dried conidia may be greatest when infected adults are introduced to healthy weevils before the loss of conidia occurs. This was demonstrated in a study on *P. japonica* where the 9 day mortality fell from over 60 to 40% when exposure of fungus-infected adults to healthy beetles was delayed for 24 h^22^.

Earlier studies have suggested that conidia formed on cadavers could be a potential inoculum source and could readily deliver ongoing inoculum to the pest population^17,25–28^. In our study we tested the potential of conidiated cadavers to control live adults under two sets of experimental conditions. In the confined environment experiment we observed close physical contact between adults and conidiated cadavers and consequently high mortality was observed, around 95% (B27) and 100% (ECS1) at the 1:1 ratio and around 81% (B27 and ECS1) at the 1:20 ratio at 9 days post-introduction. However, high mortality of adults exposed to the same treatments was not observed in the insect cage experiment. Even at 12 days post-introduction, the mortality of adults inside the insect cage was only 62.5% (B27) and 27.5% (ECS1) at the ratio 1:1 and 18.8% (B27) and 3.8% (ECS1) at the ratio 1:20. Clearly these differences could be the result of the disparity in volume of the space being occupied by the insects (148 times greater in the cage), affecting the frequency and duration of contact between healthy, infected and dead weevils and hence the transmission of conidia. Differences in relative humidity (RH) in the test environments may also be involved.

A similar study to ours found that the corrected mortality of healthy *D. citri* at 10 days post-introduction to *B. bassiana* conidiated cadavers at the ratios 1:2 and 1:20 in 500 mL containers was around 70% and 39% respectively, whereas under field conditions the corresponding mortalities of initially healthy *D. citri* were reduced to only 48% and 17%^26^. The response of *D. citri* to *Isaria fumosorosea* Wize conidiated cadavers was also evaluated where the corrected mortality was around 56% and 24% at the ratios of 1:2 and 1:20 respectively in 500 mL containers and 47% and 7% respectively in the field^26^.

Relative humidity (RH) is a major factor influencing the successful use of fungal entomopathogens as pest control agents^9,10^. Our insect cages were maintained at 56% RH in the insectary, whereas the Conviron A1000 growth chamber used to house the 500 mL containers was maintained at 65% RH. Some studies have shown that slight increases RH can improve the activity of fungal entomopathogens on their hosts^37–40^. By increasing RH from 50 to 70% mortality of the coffee berry borer *Hypothenemus hampei* Ferrari previously infected with *B. bassiana* at 1 × 10^6^ conidia/mL increased from 69 to 87%^40^. By increasing RH from 60 to 70% mortality of apple rust mite (*Aculus schlechtendali* Nal.) increased from 39 to 53% after treatment with *Paecilomyces lilacinus* (Thom) Samson at 1 × 10^5^ conidia/mL and from 76 to 89% after treatment at 1 × 10^8^ conidia/mL^39^.

Although the mortality of weevils inside the insect cages was not as high as in the 500 mL containers, there was clear evidence that after 12 days of the experiment live adults all had attached fungal conidia from the cadavers. At this time all live adults from the insect cages were incubated at high humidity (> 95%) for 24 h. The mortality of initially healthy adults 5 days later increased to around 77.5% (for B27) and 50% (for ECS1) at the ratio 1:1 and around 25% (for B27) and 6.25% (for ECS1) at the ratio 1:20 (Fig. 3). SEM evidence showed that at the 1:5 ratio all adults had physical contact with conidiated cadavers based on the high number of conidia on hairs on the tarsal pad and tibial comb (Fig. 6). These infected adults subsequently contacted other adults, as shown by the conidia found on the elytra and pronota of other individuals (Fig. 7). High densities of conidia were also found on the compound eyes and rostrums (Fig. 5), suggesting that infected adults used their forelegs to which conidia are attached to groom these body parts. Overall, our results suggest that while the weevils inside the cage had numerous attached conidia acquired via physical contact with conidiated cadavers or fungus-infected adults, the conidia could not germinate and infect adults quickly when the RH was below a certain level.

Between *B. bassiana* strain B27 and *M. anisopliae* strain ECS1, we often found that the conidia of B27 germinated on the weevil's cuticle at 6 days post-introduction whereas ECS1 conidia germinated at 9 days. This suggests that *M. anisopliae* conidia may be more sensitive to low RH than those of *B. bassiana*. Supporting this theory, an earlier study found that conidia of all tested strains of *B. bassiana* germinated faster and with higher total percentage germination than most strains of *M. anisopliae* when the incubation conditions were unfavourable (water activity was around 0.93 a_w_)^41^. When the water activity was high (> 0.99 a_w_) the conidia of most strains of *M. anisopliae* germinated faster and with higher total percentage germination than strains of *B. bassiana*^41^.

In this study adult weevils did not show avoidance behaviour towards conidiated cadavers killed by either *B. bassiana* or *M. anisopliae*. This is supported by the results of the SEM investigation where fungal conidia were found on all the specimens examined, and is similar to the results of other studies, where coleopteran species showed no avoidance behaviour toward *B. bassiana*^27,28,42^. However, our results contrast with some studies where coleopterans showed avoidance behaviour toward both *M. anisopliae*^43–45^ and *B. bassiana*^46^. A recent study has shown that *M. anisopliae* is able to produce volatile organic compounds (1-octen-3-ol, 2-octen-1-ol, 3-octanol, 3-octanone) and acetic acid. These compounds act as repellents for many insect species such as *C. formicarius*^43^, *P. japonica*^44^ and groundnut bruchid, *Caryedon serratus* Olivier^45^. *Beauveria bassiana* however, is not capable of producing these compounds^47^. The reported deterrent effect of *B. bassiana* on *O. sulcatus* could be the result of formulation additives rather than the entomopathogen itself^46^. A study similar to ours showed that the presence of *B. bassiana* conidiated cadavers on the topsoil may result in horizontal infection to *L. decemlineata* in open environments^27^. The adults of *L. decemlineata* did not show any avoidance behaviour toward the cadavers and they tended to have higher infection levels when the number of conidiated cadavers on the topsoil was increased^27^. Our results also show that the number of adults infected by *B. bassiana* can be increased by increasing the proportion of cadavers on the seedlings relative to the number of healthy weevils present (Fig. 4A). The lack of avoidance behaviour in our study could be due to the production of only non-repellent volatiles by strain ECS1, the complete absence of volatile production in this strain, or the dilution of volatiles by increased air movement in the insect cages. Further work could investigate volatile production by ECS1 and incorporate olfactometer studies to further assess the effect of any volatiles produced on *K. macadamiae* behaviour under controlled conditions.

In this study we have demonstrated that fungal entomopathogens could provide an additional means of sustainable control of adult weevils through horizontal transmission from fungus-infected adults to healthy adults and horizontal infection arising as a consequence of physical contact with conidiated cadavers. During the period of *K. macadamiae* activity between September and December temperatures are < 27 °C with RH of 65–75% (Supplementary Fig. 1)^48^, suggesting that the entomopathogens could be very effective in the orchard at this time. We believe that the microclimate in the macadamia orchards is more suitable for the fungal entomopathogens than indicated by the data from the nearest meteorology station (lower temperatures and higher RH). This assumption is based on the thick foliage and dense shade within the canopies of mature macadamia trees. As weather conditions in the Northern Rivers are ideal for the persistence of entomopathogenic fungi, the two strains used in this study are strong candidates for macadamia seed weevil control. Additional research is required to optimise biopesticide formulations to best suit application to tree crops and enhance fungal persistence, and to develop an attract-and-infect technique for field use.

## Materials and methods

### Insects and seedlings

*Kuschelorhynchus macadamiae* cannot currently be reared on artificial media, therefore weevil infested nuts were collected at 2 week intervals from three locations (28° 51′ 12ʺ S 153° 27′ 37ʺ E, 28° 48′ 27ʺ S 153° 25′ 23ʺ E and 28° 52′ 07ʺ S 153° 24′ 06ʺ E) between October and December 2018/2019 in the Northern Rivers. More than 9400 infested nuts were collected from these locations. The weevils were obtained from the infested nuts and fed as described in our previous study^14^.

Macadamia seedlings (approximately 30 cm in height, 4-months old, variety H2) for the studies were purchased from Next Block Nursery, Fernleigh, NSW. The seedlings were placed in the glasshouse (26 ± 1 °C and 54 ± 1% RH in the day and 21 ± 1 °C and 65 ± 1% RH at night) for at least 4 weeks before experimentation.

### Fungi

In this study two fungal strains were used, ECS1 (*M. anisopliae*) and B27 (*B. bassiana*). These strains have been lodged in the Queensland Plant Pathology Herbarium, Department of Agriculture and Fisheries, Brisbane, with accession numbers BRIP 70,272 (ECS1) and BRIP 70,267 (B27). Strain ECS1 was cultured on sterile Sabouraud dextrose agar supplemented with 1% (w/v) yeast extract (SDAY)^49^ and strain B27 was cultured on sterile malt extract agar (MEA) media^49^. All fungal strains were incubated in the dark at 25 ± 1 °C for 15 days before harvesting the conidia for experimentation.

Conidial suspensions of both fungal strains were prepared by scraping the surface of the conidiated cultures with a sterile spatula and suspending the inoculum in 10 mL of sterile Tween 20 (0.05% v/v in distilled water) in a 50 mL centrifuge tube (Labtek Pty Ltd, Brendale, Queensland). The suspensions were homogenised by vortexing for 5 min and the conidial concentrations were determined using a haemocytometer (Laboroptik Ltd, Lancing, UK) and an Olympus BX53 compound microscope (400x) equipped with a digital camera (Model DP74, Olympus Australia Pty Ltd, Macquarie Park, NSW). Conidia concentrations were then adjusted to LC_95_ levels; 2.49 × 10^7^ conidia/mL and 4.64 × 10^7^ conidia/mL for ECS1 and B27, respectively^14^. The germination of both fungal species was checked before experimentation and was always > 90%. The conidia were considered to have germinated when the germ-tubes were twice the diameter of the conidia^50^.

### Obtaining conidiated cadavers and conidia quantification

A group of ten mixed-sex adults was randomly collected from the insectary and placed in a 500 mL plastic container (9.5 cm diameter and height) with small ventilation holes (2 mm diameter) in the lid of each container. Prior to spray applications of the entomopathogens, all containers were chilled at 4 °C for 15 min to reduce weevil mobility. Each container was then opened and sprayed with 1 mL of LC_95_ conidial suspension using an X-Press It micro-atomiser (X-Press Graph-X Pty Ltd, Moorabbin, Victoria) calibrated to deposit approximately 4 × 10^4^ ± 9 × 10^3^ conidia/cm^2^ and 7.3 × 10^4^ ± 1.6 × 10^4^ conidia/cm^2^ for ECS1 and B27, respectively. After spraying, each container received a single macadamia nut and was incubated at high humidity (> 95%) in darkness for 24 h, followed by incubation at 25 ± 1 °C, 65 ± 3% RH with a 16L:8D photoperiod in a Conviron A1000 growth chamber (Conviron Asia Pacific Pty Ltd, Melbourne, Victoria). Each container was provided with a new macadamia nut (nut in husk) every second day for 12 days, and all dead weevils were removed and placed in Petri plates containing filter paper dampened with sterile distilled water and sealed with Parafilm. These plates were incubated in the dark at 25 ± 1 °C for 7 days to stimulate conidiation. Two separate containers of insects were sprayed, each with one of the fungal strains, and this was repeated 8 times (at 3-day intervals).

The conidiated cadavers from each spraying were assessed for conidial production. After 7 days of incubation, a conidiated cadaver was randomly selected from the Petri plates, dried in an oven at 35 °C for 30 min, and transferred into separate 2 mL centrifuge tube containing 1 mL of sterile Tween 20 (0.05% v/v)^14,51^. To quantify the number of conidia per cadaver, each 2 mL centrifuge tube was vortexed for 5 min to dislodge conidia from the conidiated cadaver, and then the conidia were counted using a haemocytometer and an Olympus BX53 compound microscope (400x).

### Horizontal transmission from fungus-infected adults to healthy adults

In this experiment we examined the effect of inoculum transfer from fungus-infected adults which served as donors to healthy adults which served as recipients and determined how infection rates were driven by the proportion of the donors relative to the recipients. To confirm the potential of inoculum transfer, seven treatments were used for each fungal species (Table 1: Bioassay I). Donor weevils were marked on their elytra or pronotum with permanent red pen which was allowed to dry for 1 h so they could be easily differentiated from recipients. Three separate containers which each contained 10 marked weevils were sprayed with 1 mL of each fungal strain at the LC_95_ conidial concentration using an X-Press It micro-atomiser, fed a macadamia nut and incubated at > 95% RH for 24 h before further experimentation in each replicate. All donors were introduced to groups of recipients in a 500 mL container according to their ratios. All containers were incubated as previously described in a Conviron A1000 growth chamber. All insects were fed as described above for 12 days and dead weevils were removed daily and verified for fungal infection as previously described. This experiment was replicated eight times (at 3-day intervals) and a total of 1408 insects were used (704 adults for each fungal species).

**Table 1:** Summary of treatments used in bioassays on *Kuschelorhynchus macadamiae* in the laboratory.

Treatments	Bioassay I		Bioassays II & III	
	Donors^a^	Recipients	Donors^b^	Recipients
Control	–	10	–	10
1:1	5	5	5	5
1:2	4	8	4	8
1:5	2	10	2	10
1:10	1	10	1	10
1:20	1	20	1	20
Positive control	12	–	–	–

^a^The number of fungus-infected adults used as donors in the experiment. The fungus-infected adults were initially painted with permanent red pen ink and infected with fungal suspension at their LC_95_ conidial concentrations, followed by high humidity incubation (> 95% RH) for 24 h before being introduced to the recipients.

^b^The number of conidiated cadavers used as the donors in the experiments.

### Horizontal infection from conidiated cadavers to healthy adults in a confined environment

In this study we examined the potential for conidia transfer from conidiated cadavers to healthy adults. To confirm conidial transfer, a control and five different ratios of conidiated cadavers and healthy adults were used for each fungal species (Table 1: Bioassay II). For each ratio, healthy adults and conidiated cadavers were placed in a 500 mL container. All insects were incubated and fed as described in the previous experiment for 9 days. Dead weevils were removed daily and verified for fungal infection as described in the previous experiment. This experiment was replicated four times (at 3-day intervals) and a total of 504 healthy adults were used (252 adults for each fungal species).

### Horizontal infection from conidiated cadavers to healthy adults in an insect cage

A macadamia seedling was placed inside a Bugdorm insect rearing cage (32.5 × 32.5 × 70 cm, Australian Entomological Supplies Pty Ltd, South Murwillumbah, NSW) inside the insectary (25 ± 1 °C, 56 ± 1% RH and 16L:8D photoperiod). Conidiated cadavers killed by ECS1 or B27 were placed on the macadamia leaves (the 2nd to 5th leaves counted from the 1st bottom leave) at different ratios (Table 1: Bioassay III) without the use of pins or adhesives. After 1 h the required number of healthy adults were released into the insect cages. Dead weevils were removed daily for 12 days and verified for fungal infection as described in the previous experiment. As adult weevils killed the seedling by defoliation and ring barking after 12 days, live adults were then transferred to 500 mL plastic containers, incubated at high humidity (> 95%) in the darkness for 24 h, followed by incubation in the insectary. Weevils in each container were provided with a new macadamia nut every second day for another 5 days. This experiment was replicated 4 times (at 3-day intervals) and a total of 504 initially healthy adults were used (252 adults for each fungal species).

### Scanning electron microscopy observations on the horizontal infection of healthy adults from conidiated cadavers in insect cages

In the scanning electron microscopy (SEM) studies our aim was to identify the external body parts of the adults which had come into contact with conidiated cadavers on a macadamia seedling. Two conidiated cadavers (ECS1 or B27) were placed on two macadamia leaves (between the 2nd and 5th leaves counted from the 1st bottom leaf) of a seedling previously placed inside a Bugdorm insect rearing cage (32.5 × 32.5 × 70 cm). After 1 h, ten adults were released inside the insect cage. Four insect cages for each fungal species were used and assigned for the post release periods of 3, 6, 9 and 12 days. In total, 80 adults were used, 40 adults for each fungal species. All the insect rearing cages were maintained in the insectary for the duration of the experiment. After 3, 6, 9 and 12 days post release, all adults in each assigned cage were collected and directly fixed in 4% glutaraldehyde in 0.05 M phosphate buffer (pH 7.3) and stored at 4 °C. Five of ten fixed insects from each assigned cage were randomly selected and rinsed three times (10 min each) in 0.05 M phosphate buffer (pH 7.3). The samples were then dehydrated through a graded ethanol series (35%, 50%, 75%, 95% and 100% ethanol) with 15 min at each step. The samples were further processed using a Autosamdri 815 series A critical point dryer (Tousimis, Rockville, MD, USA) before being mounted on stubs (25 mm diameter, ProScitech Pty Ltd, Thuringowa, Queensland) using double sided carbon tape (25 mm diameter, ProScitech Pty Ltd) and then sputter coated with gold for 1 min. Specimens were examined with a SEM Neoscope JCM-6000 (JEOL Australiasia Pty Ltd, Frenchs Forest, NSW). The number of conidia was estimated from five photos of each body part at 600 × magnification.

### Statistical analysis

All analyses were performed using RStudio^52^ Version 1.2.1335. built on R^53^ Version 3.5.2. Before analyses the mortality of healthy adults was corrected using Abbott’s formula^54^ and the mortalities in the corresponding controls. Corrected data were assessed using the Shapiro–Wilk Test for normality^55^ and Levene’s Test for homogeneity of variance using the CAR (Companion to Applied Regression, Version 3.0-3) package^56^.

As the data could not be normalised by transformation, a non-parametric analysis of variance was performed. The multifactorial “F1-LD-F1” non-parametric analysis of longitudinal data in factorial experiments was used to analyse the corrected mortality of healthy adults (recipients) caused by different ratios of fungus-infected adults or conidiated cadavers (donors) over 3 repeated measures in bioassay I (6 days, 9 days and 12 days post-introduction), 3 repeated measures in bioassay II (3 days, 6 days and 9 days post-introduction) and 4 repeated measures in bioassay III (6 days, 9 days 12 days and 18 days post-introduction). Wald-type statistics (WTS) were calculated using the nparLD (Nonparametric analysis of Longitudinal Data, Version 2.1) package^57^ to check for significant effects of the ratios, repeated measures and/or their interactions (*P* < 0.05), and the pairwise Wilcoxon rank-sum test was used to separate means. The datasets for B27 and ECS1 were analysed separately. The ggplot2 (Grammar of Graphics, Version 3.2.1) package was used to generate the figures^58^.

Since the datasets at 9 days post-introduction were available for all bioassays, the relationships between the proportion of fungus-infected adults or conidiated cadavers and the mortality of healthy adults were determined. The relationships were analysed with functions *drm()* and *mselect()* of the DRC (Dose–Response Curves, Version 3.0-1) package^59^ in order to find the best fitted models by comparing the log-likelihood values, Akaike’s Information Criteria (AIC), lack of fit and residual variance of all models was evaluated against linear, quadratic and cubic regression models. All datasets were fitted to the non-linear 2-parameter log-logistic model (LL.2), $y= \frac{1}{1+\mathrm{exp}(b*\left(\mathrm{log}\left(x\right)-\mathrm{log}\left(e\right)\right))}$ , except the dataset for adult mortality caused by ECS1 cadavers inside insect cages which was fitted to the non-linear 3-parameter log-logistic model (LL.3),$y= \frac{d}{1+\mathrm{exp}(b*\left(\mathrm{log}\left(x\right)-\mathrm{log}\left(e\right)\right))}$. For both models, *d* is the upper limit, *b* is the slope, *e* is the median effective pressure (EP50) and *x* is the proportion of cadavers or donor adults.

## Supplementary Information

Supplementary Information 1.

Supplementary Information 2.

## Data Availability

All raw and processed data for this study are provided as a supplementary file.
