# Supplementary material for: Transmission of Metarhizium anisopliae and Beauveria bassiana to adults of Kuschelorhynchus macadamiae (Coleoptera: Curculionidae) from infected adults and conidiated cadavers
Source: Sci Rep. 2021 Jan 26;11:2188. doi: 10.1038/s41598-021-81647-0 (PMC7838307; doi:10.1038/s41598-021-81647-0)
Supplement: Supplementary file 2 — Supplementary Information 2. [file 41598_2021_81647_MOESM2_ESM.docx]

Transmission of *Metarhizium anisopliae* and *Beauveria bassiana* to adults of *Kuschelorhynchus macadamiae* (Coleoptera: Curculionidae) from infected adults and conidiated cadavers

Kim Khuy Khun ^a,b,*^, Gavin J. Ash ^b^, Mark M. Stevens ^c,d^, Ruth K. Huwer ^e^, Bree A.L. Wilson ^b^

^a^ Faculty of Agronomy, Royal University of Agriculture, P.O. Box 2696, Dangkor District, Phnom Penh, Cambodia

^b^ Centre for Crop Health, Institute for Life Sciences and the Environment, University of Southern Queensland, Toowoomba, Queensland 4350, Australia

^c^ NSW Department of Primary Industries, Yanco Agricultural Institute, Yanco, New South Wales 2703, Australia

^d^ Graham Centre for Agricultural Innovation (NSW Department of Primary Industries and Charles Sturt University), Wagga Wagga, New South Wales 2650, Australia

^e^ NSW Department of Primary Industries, Wollongbar Primary Industries Institute, Wollongbar, New South Wales 2477, Australia

# ^*^ Correspondence and requests for materials should be addressed to K.K.K. (email: [Khun.KimKhuy@rua.edu.kh](mailto:KimKhuy.Khun@usq.edu.au) or KimKhuy.Khun@usq.edu.au), Currently, K.K.K is at Centre for Crop Health, Australia

**Supplementary figure 1**: Mean monthly temperature, relative humidity and rainfall at the Centre for Tropical Horticulture in Alstonville, Northern Rivers, between 1991 and 2011 (compiled data from Australian Bureau of Meteorology)
